# Efficient Production of Swine Influenza Virus Using Serum-Free Suspension MDCK Cells for Vaccine Manufacturing

**DOI:** 10.4014/jmb.2606.06029

**Published:** 2026-08-21

**Authors:** Seung Jin Koo, Yeon-Gu Kim

**Affiliations:** 1 Biotherapeutics Translational Research Center, Korea Research Institute of Bioscience and Biotechnology (KRIBB), 125 Gwahak-ro, Yuseong-gu, Daejeon, Republic of Korea; 2 Department of Bioprocess Engineering, KRIBB School of Biotechnology, University of Science and Technology (UST), 217 Gajeong-ro, Yuseong-gu, Daejeon, Republic of Korea; 3 Choong Ang Vaccine Laboratory Co., Ltd. (CAVAC), 1476-37 Yuseong-daero, Yuseong-gu, Daejeon, Republic of Korea

**Keywords:** Swine influenza virus, MDCK cells, Serum-free suspension culture, Bioreactor, Cell-based vaccine

## Abstract

Cell culture–based manufacturing platforms, already well-established for human influenza vaccines, are increasingly being adopted in the veterinary field as a powerful alternative to traditional embryonated hen’s eggs, providing improved scalability and safety. In this study, we established a serum-free suspension culture process using Madin–Darby canine kidney (MDCK) cells for the efficient production of swine influenza virus (SIV). To optimize SIV production, we investigated multiple customized serum-free media in shaking flask cultures and selected an optimal formulation that supported efficient viral production. When this optimized medium was applied to a bioreactor culture, it supported robust cell growth, achieving a maximum viable cell density of 3.8 × 10^6^ cells/mL prior to infection. Subsequent direct SIV infection experiments, conducted without media exchange in both shaking flask and bioreactor cultures, consistently yielded high and stable virus titers reaching a maximum log_2_ titer of 8.0, thus confirming the excellent scalability and reproducibility of the process. Moreover, the test trivalent vaccine developed in this study using the bioreactor-produced SIV antigen triggered high hemagglutination inhibition (HI) antibody titers and robust geometric mean titers (GMT) in animal models, thereby demonstrating superior immunogenicity to egg-based commercial vaccines. Taken together, these findings suggest that MDCK cell-based suspension culture platform may provide a scalable, high-yield, and reliable framework for the modern manufacturing of cell-based SIV vaccines.

## Introduction

Swine influenza virus (SIV), an influenza A virus, is a major respiratory pathogen that affects pigs and causes significant economic losses in the global swine industry [1]. Although SIV infection typically causes low mortality, it results in substantial production losses through reduced growth performance, decreased feed efficiency, and increased susceptibility to secondary bacterial infections [2, 3]. Pigs serve as important hosts for the genetic reassortment of influenza viruses, resulting in the production of diverse viral variants [4]. In Korean swine populations, the major subtypes H1N1, H1N2, and H3N2 circulate endemically, contributing to their widespread distribution across swine farms [5, 6]. These subtypes are among the most prevalent throughout the world and are commonly included in multivalent vaccines to expand the protective coverage against diverse circulating strains [7]. Besides its effect on animal health, SIV poses a potential zoonotic threat because certain strains can infect humans and have historically propelled the emergence of novel pandemic viruses, such as the 2009 H1N1 pandemic [8, 9]. Consequently, there exists a need for effective vaccination strategies targeting the predominant circulating subtypes for controlling the disease and minimizing both economic and public health risks.

Traditionally, several viral vaccines have been produced using embryonated egg-based manufacturing systems; nevertheless, this approach has inherent limitations, including restricted scalability, dependence on a continuous egg supply, and the potential presence of residual egg-derived allergens [10]. In recent years, cell-based vaccine production technologies have been increasingly adopted, thereby shifting from traditional adherent cell cultures in roller bottles or stacked flasks to scalable suspension culture systems in bioreactors [11, 12]. Furthermore, serum-free suspension culture platforms have been widely adopted for industrial-scale production to overcome the inherent constraints of adherent systems, such as surface area limitations and labor-intensive scale-up processes [13]. Moreover, optimization strategies combining suitable serum-free media with precisely controlled bioreactor operations are being intensively investigated to maximize viral yields and ensure process consistency [14]. In addition, as veterinary biologics increasingly require manufacturing standards comparable to those for human pharmaceuticals, the implementation of modern bioreactor systems has become essential to comply with these stringent current Good Manufacturing Practice requirements [15, 16].

Madin–Darby canine kidney (MDCK) cells represent the most widely used mammalian production platform for the propagation of the influenza virus due to their exceptional susceptibility to viral infection and robust performance in large-scale manufacturing [17, 18]. In particular, serum-free suspension cultures of MDCK cells are already well established for manufacturing human influenza vaccines [19] and have been successfully applied in advanced fed-batch and perfusion systems to achieve high cell densities and maximized viral yields [20]. Conversely, the application of these advanced culture platforms to veterinary influenza vaccines, particularly those targeting SIV, remains markedly limited despite the increasing demand for scalable systems [21]. Moreover, despite the widespread commercialization of suspension-adapted bioprocesses for human viral vaccines, their veterinary counterparts remain largely confined to the experimental or developmental stages [22, 23]. The direct translation of human manufacturing processes to veterinary applications is often hindered by the stringent requirements for extreme cost-effectiveness specific to the veterinary vaccine market. Hence, establishing a cost-effective and robust suspension MDCK platform tailored specifically for SIV is a crucial step toward modernizing veterinary vaccine manufacturing.

In this study, we established a serum-free suspension MDCK cell platform for the high-yield production of three major SIV subtypes: H1N1, H1N2, and H3N2. Various culture media were screened to optimize both cell proliferation and viral replication, followed by the evaluation of infection strategies in shaking flasks. The scalability of the process was further validated in a benchtop bioreactor, ensuring consistent performance for industrial applications. Finally, the harvested viral antigens were formulated into a trivalent inactivated vaccine, and its safety and immunogenicity were comprehensively evaluated and compared with a traditional egg-based vaccine.

## Materials and Methods

### Cell line and Virus Strains

Suspension-adapted MDCK cells and three major SIV subtypes, A/Sw/Kor/CAN1/04 (H1N1), A/Sw/Kor/K1/05 (H1N2), and A/Sw/Kor/CA04 (H3N2), were provided by Choong Ang Vaccine Laboratories (CAVAC; Republic of Korea). All experiments were conducted in a Biosafety Level 2 (BSL-2) facility according to established institutional safety protocols.

### Cell Culture Conditions and Virus Propagation

The MDCK cells were maintained in a customized serum-free medium supplemented with 4 mM L-glutamine (Sigma-Aldrich, USA). Shaking flask cultures were performed in 250 mL Erlenmeyer flasks (Corning, USA) with a working volume of 50 mL at 37°C, 5% CO_2_, and 110 rpm. Process scalability was evaluated using a 2 L benchtop bioreactor (Biostat^®^ B; Sartorius, Germany) with a working volume of 1 L. The bioreactor parameters were maintained at 37°C, pH 7.2 (± 0.03), and 50% dissolved oxygen (DO) saturation, with an agitation speed of 100–150 rpm.

For the virus production, the cells were inoculated at a density of 3.0 × 10^6^ cells/mL with a 1% (v/v) viral inoculum (128 HAU/50 μL). Trypsin obtained from bovine pancreas (Sigma-Aldrich) was added to the culture at a final concentration of 1 μg/mL to facilitate viral replication. To optimize yields, the following two infection strategies were compared: direct infection and infection after medium replacement. Samples were collected every 24 h post-infection and stored at -70°C for titration. The concentration and viability of cells were determined using the trypan blue dye exclusion method using a hemacytometer (Sigma-Aldrich). A minimum cell viability of 95% was ensured before all infection experiments.

### Analytical Assays for Hemagglutination and Hemagglutination Inhibition

Virus titers were determined by a hemagglutination (HA) assay in 96-well U-bottom plates (Corning). Briefly, 50 μL of virus samples was serially diluted twofold in phosphate-buffered saline, followed by the addition of 50 μL of 1% chicken red blood cells (cRBCs). The plates were then incubated at room temperature for 30 min, and the HA titer was expressed as the reciprocal of the highest dilution exhibiting complete HA.

For immunogenicity evaluation, hemagglutination inhibition (HI) titers were measured from collected sera. Prior to the assay, serum samples were treated with a receptor-destroying enzyme (RDE-SEIKEN; Japan) at 37°C for 18 h, followed by heat inactivation at 56°C for 30 min to remove nonspecific inhibitors. HI titers were defined as the reciprocal of the highest dilution inhibiting HA, and geometric mean titers (GMTs) were calculated for each group.

### Virus Inactivation and Validation

Harvested SIV was inactivated by adding 0.2% (v/v) formalin (Merck, Germany) and incubating at room temperature (20°C–25°C) for 4 days. The inactivation was validated through two serial passages in 11-day-old specific-pathogen-free (SPF) embryonated chicken eggs. Nine formalin-treated samples (comprising triplicate independent samples for each of the three SIV subtypes: H1N1, H1N2, and H3N2) along with one uninfected negative control were inoculated into 10 individual SPF eggs (0.2 mL per egg) via the allantoic cavity and incubated for 5 days at 37°C. Subsequently, the allantoic fluid was harvested from each egg and re-inoculated into a new set of 10 SPF eggs under identical conditions for a second serial passage (5 days at 37°C). Untreated virus samples (before formalin inactivation) were maintained separately in storage to serve as baseline controls for initial viral titers. After the second passage, embryonic development was examined, and the harvested allantoic fluid along with the stored untreated virus samples was analyzed by an HA assay using 1% cRBCs.

### Preparation of Test Vaccines

Three experimental vaccine groups were prepared, viz., two cell-based formulations, including cell-based test vaccine #L (666 HAU/subtype/dose) and cell-based test vaccine #H (1280 HAU/subtype/dose), and one egg-based commercial vaccine as a positive control. Both cell-based formulations contained uniform antigen levels for each of the three SIV subtypes (H1N1, H1N2, and H3N2). The egg-based vaccine was derived from a commercial SIV vaccine (SuiShot^®^ Flu-3; CAVAC), which contained nominal antigen levels of >2,000 HAU/dose for each subtype. All vaccine formulations, including the cell-based test vaccines and the commercial egg-based reference, were prepared under identical conditions using a standardized oil-free nanoparticle adjuvant at a final concentration of 25%. All the vaccine formulations were stored at 4°C before conducting animal experiments.

### Animal Safety and Immunogenicity Tests

For safety test, eight guinea pigs and eight mice per experimental group (cell-based test vaccines #L, #H, and egg-based vaccine) were monitored. According to the official testing standards for SuiShot^®^ Flu-3, guinea pigs received 2.0 mL via intramuscular (IM) and intraperitoneal (IP) routes, whereas mice received 0.5 mL via the IP route. The animals were monitored for 7 days for clinical signs, body weight changes, and mortality. For the immunogenicity test, each experimental group consisted of seven guinea pigs that were randomly assigned and immunized twice at a two-week interval via the intramuscular route with a final injection volume of 2.0 mL. Serum samples were collected two weeks after the second vaccination, and the HI titers against the three SIV subtypes were measured and compared with those of the negative control group. All animal experiments were performed in accordance with the guidelines and approved by the Institutional Animal Care and Use Committee (IACUC) of CAVAC (Approval Number: 230623-07).

### Statistical Analysis

Experimental results are expressed as mean ± standard deviation (SD). *In vitro* cell culture and *in vivo* experiments were performed in triplicate (*n* = 3) and duplicate (*n* = 2), respectively. Statistical significance between two experimental groups was analyzed using an unpaired Student’s *t*-test. For comparisons among three or more groups, a one-way analysis of variance (ANOVA) followed by Tukey’s multiple comparisons post hoc test was performed. A *p*-value of < 0.05 was considered statistically significant.

## Results and Discussion

### Screening of Serum-Free Media for Efficient SIV Production and MDCK Cell Growth

**Fig. 1:**
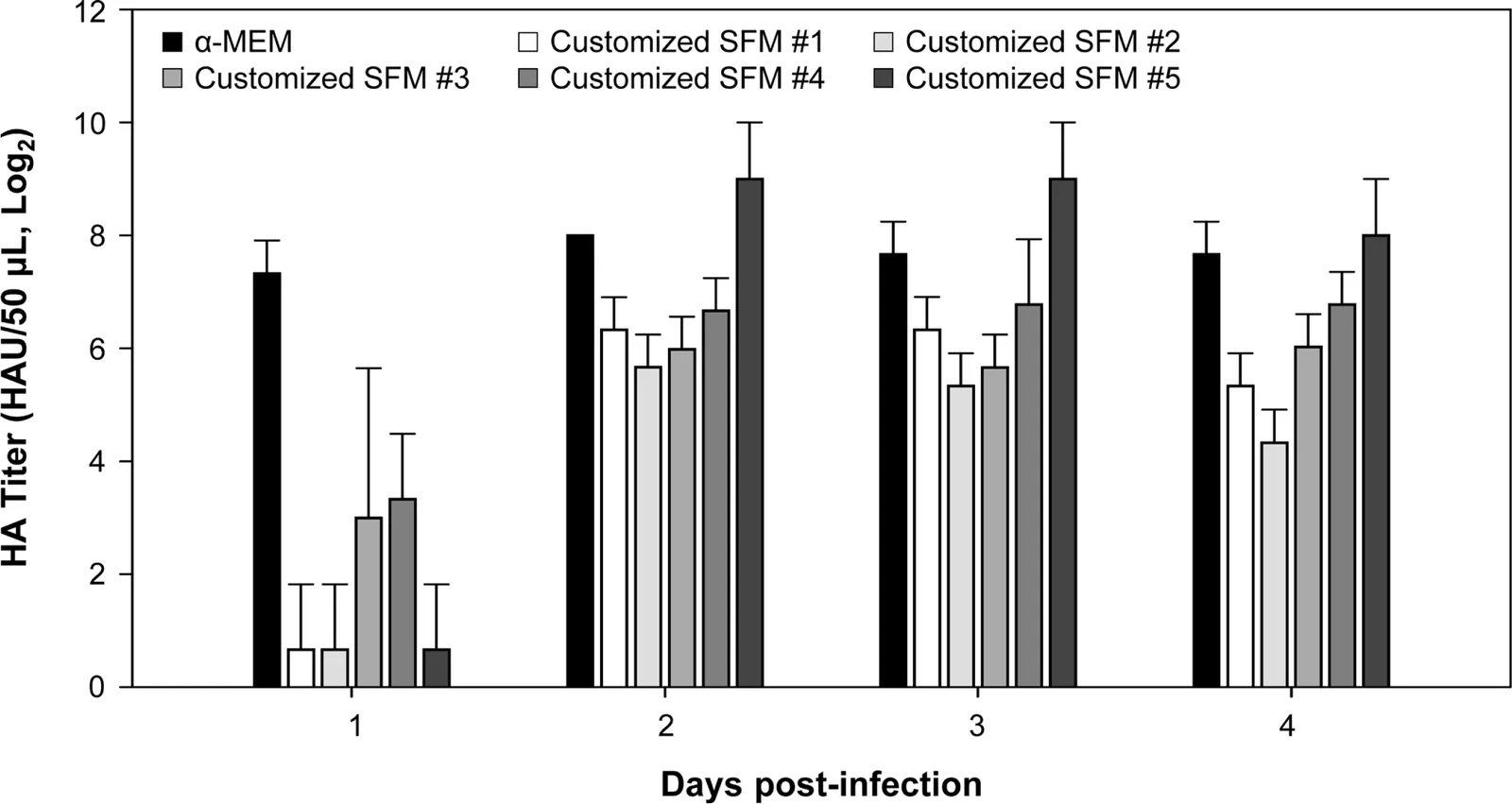
Profiles of swine influenza virus (SIV) production in MDCK cells after medium exchange into various culture media. Suspended cells initially maintained in a serum-containing medium (α-MEM + 10% FBS) were subjected to complete medium replacement with either five customized serum-free media (Customized SFM #1–#5) or basal α-MEM as a control before infection. Cells were infected with the H1N2 subtype at a density of 2.0 × 10^6^ cells/mL with a cell viability of >95% in 250 mL shaking flasks. Virus production was monitored daily for 4 days post-infection (dpi) by a hemagglutination (HA) assay. Error bars represent standard deviations calculated from biological triplicate experiments (*n* = 3). Statistical significance across the data obtained on different culture media at 2 dpi was determined using a one-way ANOVA followed by Tukey’s multiple comparisons test (*p* < 0.05 for Customized SFM #5 vs. Customized SFM #1–4; *p* > 0.05 for Customized SFM #5 vs. α-MEM).

The composition of serum-free media strongly influences cellular metabolism, membrane composition, and host–virus interactions, thereby affecting the efficiency of viral replication in mammalian cell cultures [24]. Therefore, selecting an appropriate medium is a crucial step in developing scalable cell-based vaccine manufacturing processes. In the present study, to identify an optimal serum-free medium for efficient SIV production, five different customized serum-free media (SFM) formulations along with serum-containing medium as a control were investigated using suspension-adapted MDCK cells in 250 mL shaking flasks. The cells were infected with the H1N2 subtype at a density of 2.0 × 10^6^ cells/mL with viability >95% to minimize variations originating from cell physiological status. The production of virus was monitored daily for 4 days post-infection (dpi) using an HA assay (Fig. 1).

As shown in Fig. 1, the HA titers increased rapidly after infection and reached peak levels at 2–3 dpi in the majority of the tested media. Among the examined conditions, customized serum-free medium #5 supported the highest virus production, reaching approximately 2^9^ HAU/50 μL (log_2_ titer 9.0) at 2 dpi. Notably, the viral yield obtained using customized SFM #5 at this time point was significantly higher than that in the other tested SFM (*p* < 0.05) and was statistically comparable to that of the serum-containing control (*p* > 0.05). The steady increase in viral titers indicates that the customized serum-free medium #5 efficiently supports active viral replication and also maintains adequate host cell viability during the critical production phase. Specifically, customized SFM #5 has been rationally designed to enhance cell growth by optimizing the balance of amino acids, vitamins, lipids, trace elements, and other growth-promoting components. Because it is a complex mixture formulated through multi-component statistical optimization, identifying the specific impact of individual components is challenging. That being said, it is fundamentally characterized by a higher specific amino acid content compared to the other candidate media, which robustly supports high-density cell growth.

**Fig. 2:**
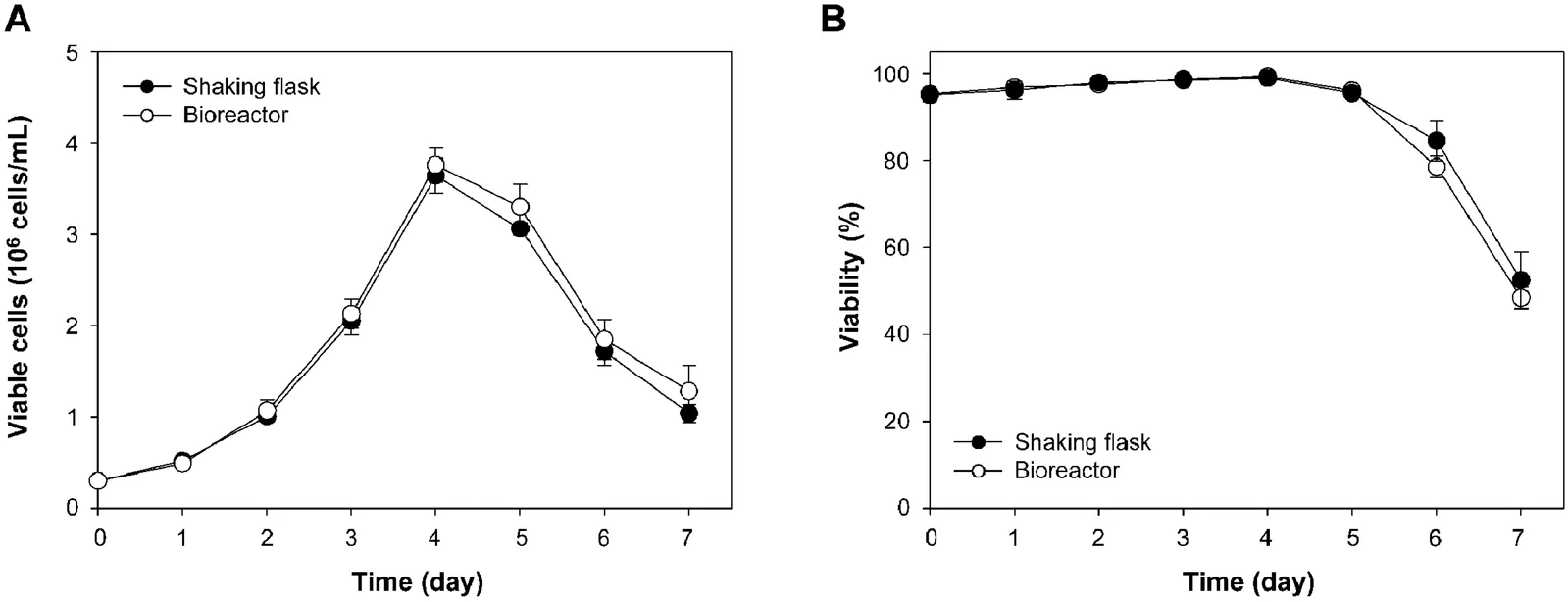
Growth profiles and viability of suspension-adapted MDCK cells in customized serum-free medium selected in Fig. 1. Suspended cells were cultivated over a 7-day period in both 250 mL shaking flasks and a 2 L benchtop bioreactor to determine the optimal time point for virus infection. (**A**) Viable cell density and (**B**) cell viability were monitored daily in both culture systems. Error bars represent standard deviations calculated from biological triplicate experiments (*n* = 3).

Following medium screening, the growth performance of MDCK cells in customized serum-free medium #5 was examined in both 250 mL shaking flasks and a 2 L benchtop bioreactor to determine optimal infection conditions (Fig. 2). As shown in Fig. 2A, the cell density increased steadily and reached 3.6 × 10^6^ cells/mL in shaking flasks and 3.8 × 10^6^ cells/mL in the bioreactor by day 4, after which the cultures entered a stationary plateau through day 7. The cell viability remained > 95% up to day 4 in both systems before gradually declining thereafter (Fig. 2B), indicating that the selected medium supports robust cell growth during the exponential phase under suspension conditions. No significant differences in growth patterns were observed between the two culture platforms, suggesting that the culture environment in the shaking flask adequately represents the bioreactor conditions.

A high cell viability before infection is particularly essential for the influenza virus production, as compromised cells often result in reduced viral yields and inconsistent antigen quality. Based on these results, virus infection was performed when the cell density exceeded 3.0 × 10^6^ cells/mL with a viability above 95% (corresponding to day 4 of culture); these conditions are considered favorable for efficient virus replication and antigen production.

### Production Characteristics of Three SIV Subtypes in Shaking Flasks and a Benchtop Bioreactor

**Fig. 3:**
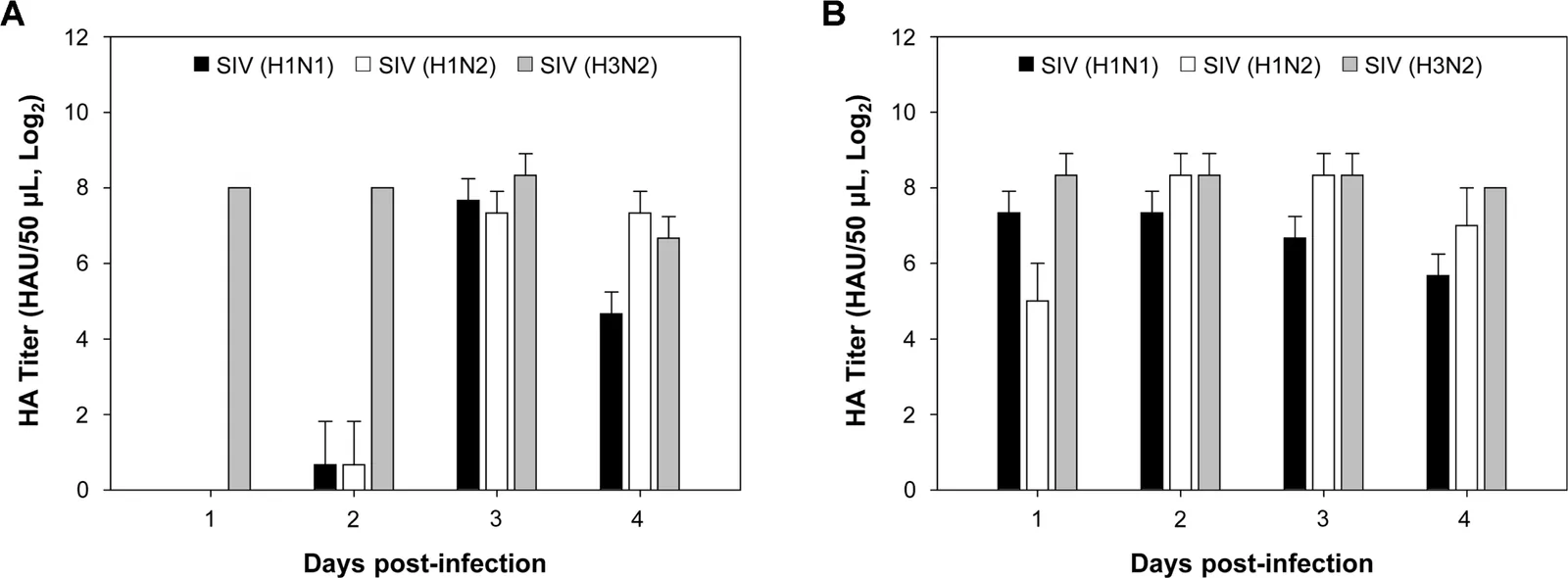
Profiles of virus production for three swine influenza virus (SIV) subtypes under different infection strategies in shaking flask cultures. Suspended MDCK cells were infected with H1N1, H1N2, or H3N2 subtypes in 250 mL shaking flasks using the customized serum-free medium selected in Fig. 1, and log_2_ HA titers were monitored daily for 4 days post-infection (dpi). Two distinct operational strategies were examined and compared: (**A**) direct infection without medium exchange and (**B**) infection after complete medium replacement with fresh serum-free medium. Error bars represent standard deviations calculated from biological triplicate experiments (*n* = 3). Statistical significance between the data from the direct infection and medium replacement strategies at specific time points was determined using an unpaired Student’s *t*-test (*p* < 0.05 for early propagation at 1–2 dpi; *p* > 0.05 for final yields at 3 dpi).

After the successful evaluation of customized serum-free medium #5, the production characteristics of three major SIV subtypes (H1N1, H1N2, and H3N2) were characterized to establish a robust and versatile manufacturing process. To streamline the operational workflow, two distinct infection strategies were evaluated in 250 mL shaking flasks: direct infection without medium exchange and infection after replacing the culture medium with fresh serum-free medium (Fig. 3).

As shown in the shaking flask experiments (Fig. 3), the two infection strategies exhibited markedly different viral replication kinetics during the initial stages. For the H1N1 and H1N2 subtypes, the medium replacement strategy (Fig. 3B) significantly promoted early viral propagation (*p* < 0.05 compared to direct infection), yielding detectable and increased HA titers at 1–2 dpi. In contrast, the direct infection method (Fig. 3A) exhibited a distinct lag phase for H1N1 and H1N2 during the first 2 days. However, by 3 dpi, the maximum HA titers from both strategies converged to statistically equivalent peak levels (*p* > 0.05), ranging consistently between 7 and 8 log_2_ HAU/50 μL across all three SIV subtypes. Notably, the H3N2 subtype showed exceptionally robust replication kinetics, reaching maximum yields (~8 log_2_ HAU/50 μL) as early as 1 dpi regardless of the infection strategy or the presence of spent medium. The differences in the propagation efficiency among these viruses are most likely due to inherent strain-specific biological variations, such as superior receptor-binding affinity or enhanced polymerase activity in MDCK cells, which are frequently observed among different virus strains.

The accelerated early viral replication observed with the medium replacement strategy can be attributed to the replenishment of essential nutrients and the removal of accumulated inhibitory metabolites. Conversely, the initial lag phase seen in the direct infection method is most likely due to the suboptimal nutritional conditions provided by the spent medium. From an industrial bioprocess perspective, medium replacement requires labor-intensive, multi-step unit operations such as cell sedimentation or centrifugation, which introduce higher risks of contamination, cell loss, and increased manufacturing costs at larger scales. As no significant differences were observed in the final antigen yields at 3 dpi between the two strategies (*p* > 0.05), the direct infection method was selected as the final optimized process as it was easier to operate, cost-effective, and scalable.

**Fig. 4:**
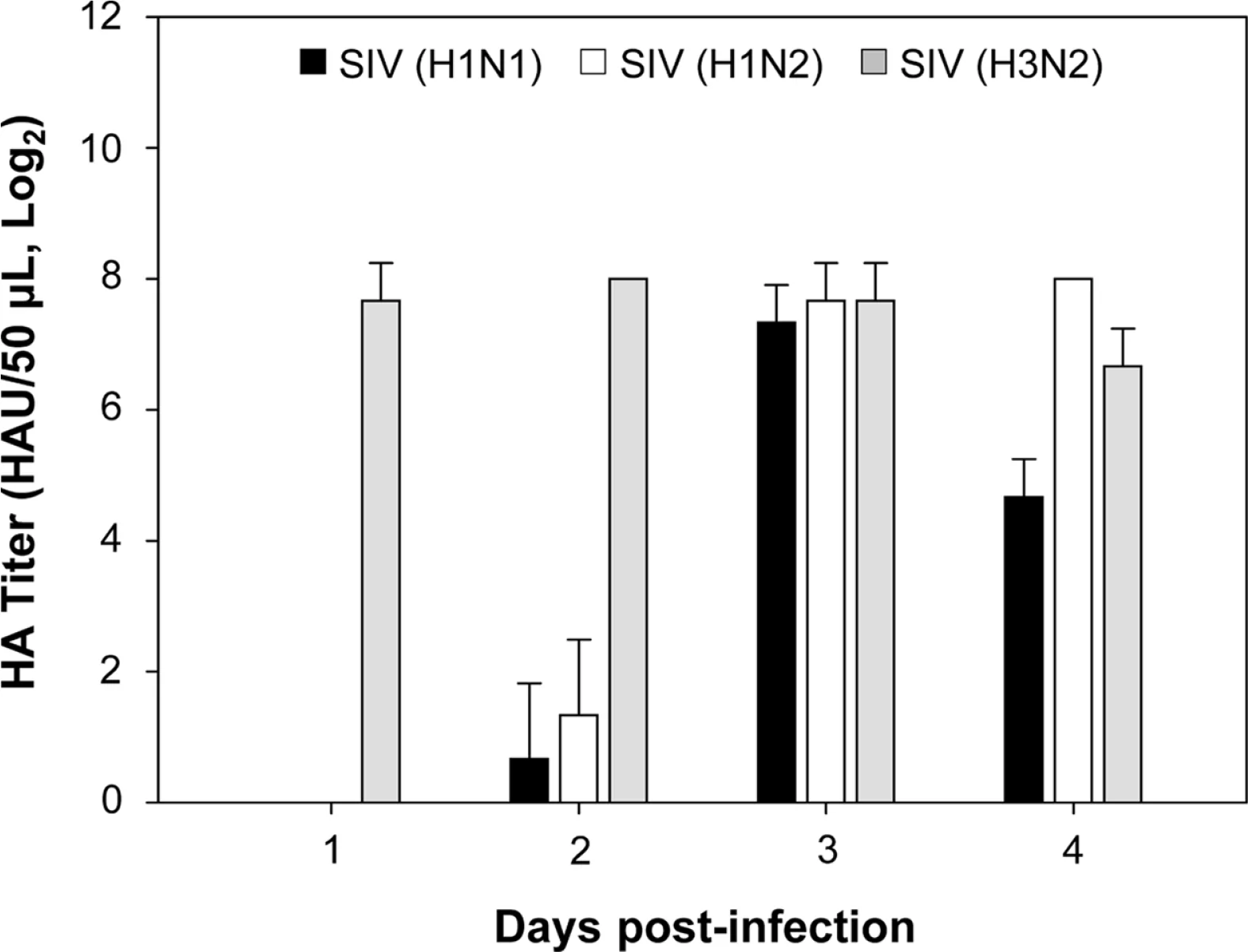
Virus production profiles of three swine influenza virus (SIV) subtypes in a 2 L benchtop bioreactor. Suspended MDCK cells were cultured in the customized serum-free medium selected in Fig. 1 and infected using the optimized direct infection strategy without medium exchange. Log_2_ HA titers for H1N1, H1N2, and H3N2 subtypes were monitored daily for 4 days post-infection (dpi). Error bars represent standard deviations calculated from biological triplicate experiments (*n* = 3).

Furthermore, to validate the scalability of the selected direct infection process, cultivation was scaled up to a 2 L benchtop bioreactor using customized serum-free medium #5 (Fig. 4). The subtype-specific production profiles and viral kinetics in the bioreactor were in excellent agreement with the patterns observed in the shaking flask experiments. Following direct infection, the HA titers for H1N1 and H1N2 remained low during 1–2 dpi but increased sharply to reach peak plateaus of 7–8 log_2_ HAU/50 μL at 3 dpi (Fig. 4). The H3N2 subtype maintained its characteristic rapid replication behavior, exhibiting high and stable titers throughout the early cultivation period before experiencing a minor decrease at 4 dpi. The successful reproduction of these kinetic trajectories and peak yields at the 2 L bioreactor scale demonstrates that the direct infection method developed at the flask level is highly reliable, robust, and readily scalable. These findings firmly establish that a 72 h post-infection period (3 dpi) serves as the optimal harvest time point to maximize volumetric antigen productivity along with preventing potential product degradation in large-scale culture vessels.

### Validation of the Inactivation of SIV Antigens

To determine the safety and verify the complete loss of the replicative capacity of the inactivated SIV (H1N1, H1N2, and H3N2) antigens, the nine formalin-treated viral preparations, along with an uninfected negative control, were inoculated into 11-day-old SPF embryonated chicken eggs for a two-passage validation protocol. During both the first and second serial blind passages, all the 10 eggs showed no macroscopic signs of viral replication or tissue lesion formation, testing entirely negative.

**Fig. 5:**
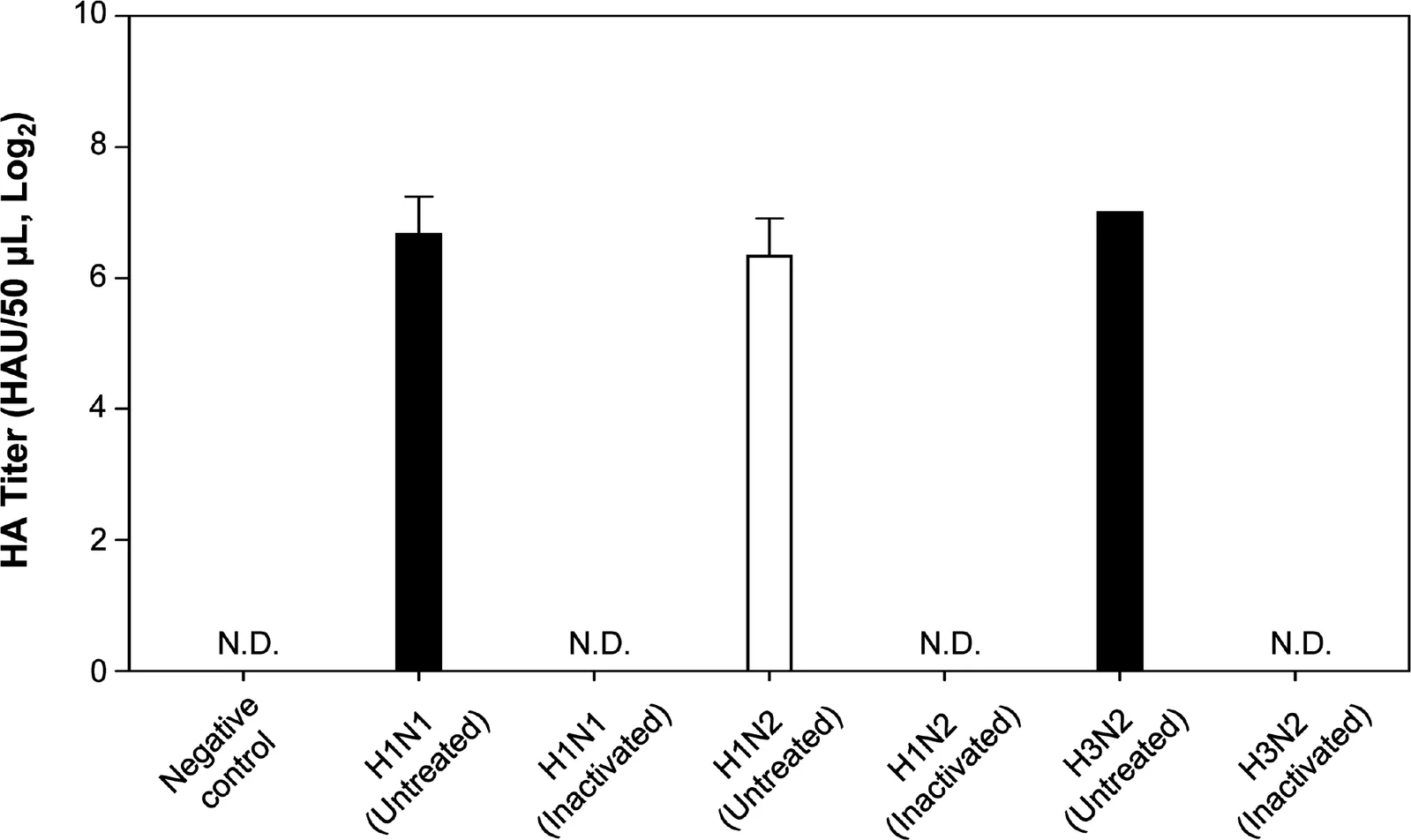
Evaluation of formalin inactivation efficacy and safety for cell-cultured swine influenza virus (SIV) antigens. Comparison of log_2_ HA titers was performed between initial untreated virus stocks (Untreated) and formalin-treated antigen preparations (Inactivated) after two serial blind passages in 11-day-old SPF embryonated chicken eggs. Untreated antigens of H1N1, H1N2, and H3N2 subtypes were analyzed along with their corresponding inactivated counterparts and an uninfected negative control. Error bars represent standard deviations calculated from biological triplicate experiments (*n* = 3). N.D., not detected.

Fig. 5 compares the HA titers of the initial untreated virus stocks with those of the post-passage allantoic fluids to quantitatively demonstrate the efficacy of the inactivation process. The initial untreated virus samples (which did not undergo egg passages) showed robust baseline HA activity, yielding log_2_ HA titers of 6–7 across all subtypes, thus confirming high starting viral loads before treatment. In sharp contrast, after the 4-day formalin treatment and the subsequent two serial passages in eggs, all the corresponding post-treatment samples across all three subtypes, as well as the uninfected negative control, demonstrated a complete absence of HA activity, yielding a definitive log_2_ HA titer of 0. No embryonic death or abnormal lesion formation was observed, thereby confirming that the viral replicative competency was thoroughly and successfully abolished by the formalin treatment. Furthermore, gross pathological autopsies of the inoculated embryonated eggs revealed no inflammatory reactions, tissue necrosis, or hemorrhagic spots within the mucosal or vascular structures. These findings confirm that the optimized formalin inactivation process is highly effective and that the resulting monovalent antigen bulks are devoid of residual virulence and acute tissue toxicity, thus establishing their safety for the downstream trivalent vaccine formulation.

### Safety and Immunogenicity of the Trivalent SIV Vaccine

**Fig. 6:**
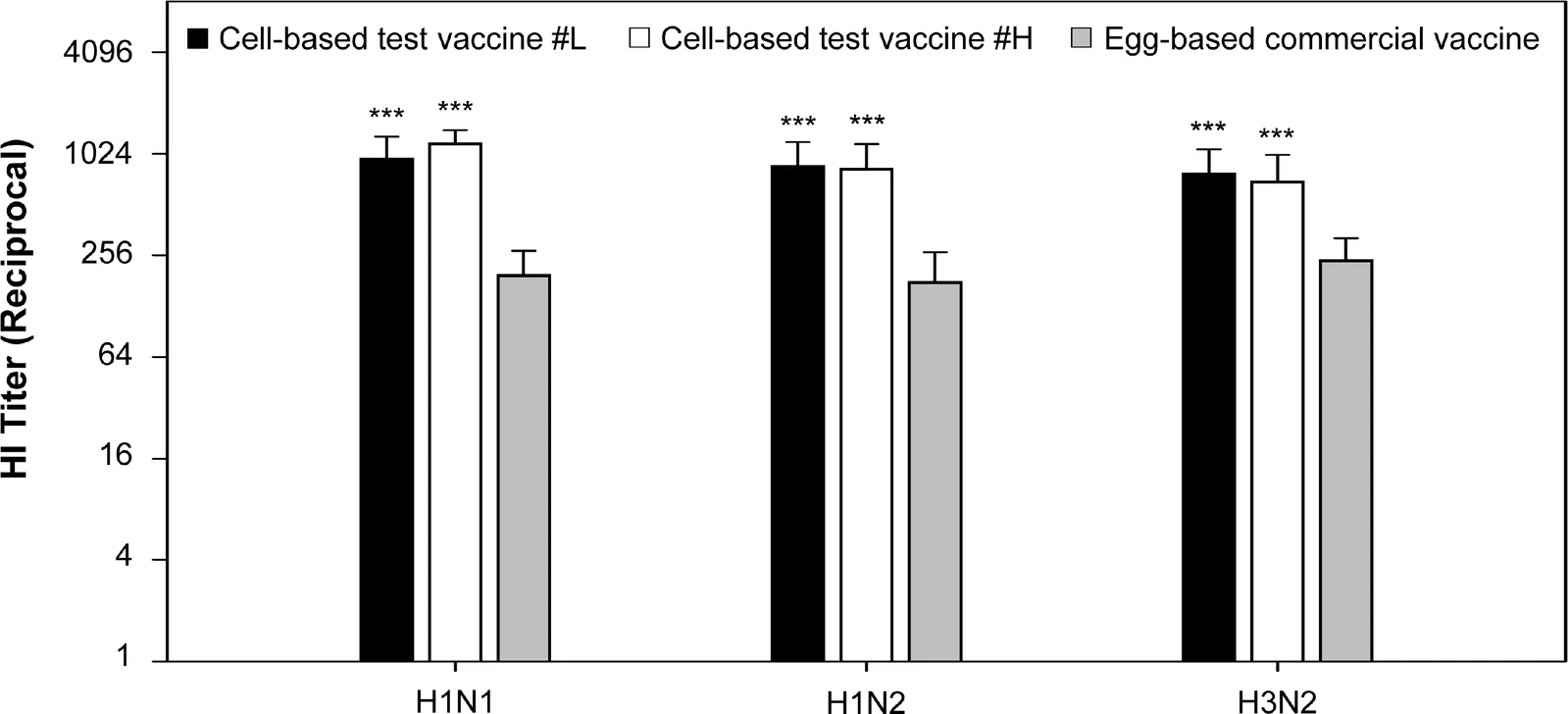
Hemagglutination inhibition (HI) antibody titers against three swine influenza virus (SIV) subtypes after *in vivo* immunization with trivalent vaccine formulations in guinea pigs. Serum samples were collected from guinea pigs two weeks after the final vaccination to measure specific humoral immune responses against H1N1, H1N2, and H3N2 subtypes. Immune responses induced by cell-based test vaccine #L (black bars) and cell-based test vaccine #H (white bars) were compared directly with those of the egg-based commercial vaccine (gray bars). Error bars represent standard deviations calculated from technical triplicates of biological duplicate experiments (*n* = 2). Asterisks indicate a statistically highly significant difference (***p < 0.001) compared with the traditional egg-based commercial vaccine group.

Following the preparation of the trivalent SIV vaccines—two cell-based formulations (cell-based test vaccine #L and #H) and one traditional egg-based comparator (egg-based commercial vaccine)—we rigorously investigated their *in vivo* safety. During the 7-day post-vaccination observation period, no mortality, abnormal clinical signs, or notable alterations in body weight and physical activity were observed in either guinea pigs or mice. These outcomes indicate that all the trivalent vaccine formulations were well tolerated and did not induce acute systemic toxicity. The immunogenicity of the trivalent vaccines was evaluated by measuring hemagglutination inhibition (HI) antibody responses two weeks after the final vaccination (Fig. 6). As demonstrated across the SIV subtypes summarized in Fig. 6, both cell-based formulations induced robust HI antibody titers against the H1N1 subtype, with individual values ranging from 640 to 1280. In particular, the cell-based test vaccines #L and #H achieved GMTs of 951 and 1174, respectively, indicating a statistically highly significant increase compared with the GMT of 195 observed in the egg-based commercial vaccine group.

A similar trend of enhanced immunogenicity was observed for the H1N2 subtype (Fig. 6), where the cell-based test vaccine #L and #H groups exhibited high HI titers between 640 and 1280, yielding GMTs of 861 and 830, respectively. In contrast, the egg-based commercial vaccine group showed substantially lower responses ranging from 80 to 320 (GMT of 177). For the H3N2 subtype (Fig. 6), the HI titers elicited by the cell-based test vaccines #L and #H extended from 640 to 1280 (GMTs of 780 and 698, respectively), again clearly outperforming the egg-based commercial vaccine reference, which yielded HI titers between 160 and 320 (GMT of 238).

Importantly, the negative control group exhibited HI titers below the limit of detection (< 10) across all the tested subtypes (omitted from Fig. 6 for visual clarity), confirming that the observed humoral immune responses were highly specific to the administered antigen formulations. Collectively, these *in vivo* results demonstrate that the MDCK cell-based manufacturing platform induces significantly higher and more robust antibody responses than the traditional egg-based system across all epidemiologically relevant SIV subtypes. This enhanced immunogenicity strongly supports the feasibility and potential of using cell-based platforms as an effective alternative for the industrial production of SIV vaccines.

In this study, the scale-up to a 2 L benchtop bioreactor successfully demonstrated the feasibility and robustness of our suspension culture process for SIV production (Fig. 4). Indeed, achieving a maximum viable cell density of 3.8 × 10^6^ cells/mL in a batch culture mode without cellular aggregation represents a significant step toward replacing traditional egg-based manufacturing. However, to fulfill the stringent industrial demands for mass vaccine production and maximize cost-efficiency, further intensification of the bioreactor operating strategies is required. As demonstrated in Fig. 3, our customized SFM #5 enables direct viral infection without medium exchange, greatly simplifying the overall production process. In the future, we plan to leverage this operational advantage by developing a fed-batch strategy utilizing tailored nutrient supplementation. This approach will aim to accelerate cell growth and ultimately enhance the total viral yield. Furthermore, process intensification strategies—such as high-cell-density inoculation integrated with perfusion culture, which is representative of CHO cell-based therapeutic protein production systems can be implemented into our MDCK cell-based platform to shorten production timelines and maximize volumetric virus production [25]. Finally, beyond increasing viable cell density, maximizing specific virus productivity through culture condition optimization, including low temperature culture, osmolarity optimization, and precise pH adjustments, will be investigated in future studies to establish a high-titer commercial manufacturing framework.

Notably, the cell-based vaccines elicited substantially stronger HI antibody responses than the egg-based control, despite the latter containing higher nominal antigen doses (>2000 HAU/dose vs. 666–1280 HAU/dose). This discrepancy suggests a robust immunological potency of the cell-based platform. While our study did not directly analyze the specific antigen quality or mutational profiles of the vaccines, previous studies have reported that traditional egg-based vaccine manufacturing can introduce abundant egg-derived proteins, primarily ovalbumin, that may confound accurate antigen quantification [26, 27]. Furthermore, passage in embryonated eggs has been documented to potentially induce egg-adaptive mutations in the viral HA protein, which might alter key neutralizing epitopes [28, 29]. We hypothesize that the enhanced immunogenicity observed can be partially explained by the use of cell-based groups, thereby avoiding the risks associated with egg-based systems, such as egg-derived impurities and potential adaptive mutations. Furthermore, theoretically, serum-free suspension culture platforms could allow for better preservation of antigenic fidelity, suggesting that cell-derived antigens can elicit potent humoral responses even at lower nominal doses.

From an analytical perspective, the HA assay is the standard surrogate marker for evaluating total immunogenic antigen mass (*i.e.*, total hemagglutinin content) in inactivated vaccine manufacturing. Therefore, it is suitable for preliminary investigations of the protective efficacy of inactivated swine influenza vaccines. Nevertheless, to differentiate between infectious and non-infectious viral particles, we plan to incorporate infectivity assays, such as TCID_50_ or EID_50_ in subsequent investigations in addition to further process optimizations. This will provide a more comprehensive characterization of infectious viral replication kinetics and further validate the efficiency of our optimized platform. While the HI assay is widely established for assessing the protection conferred by influenza vaccines, we did not perform a complementary virus neutralization (VN) assay to confirm functional neutralizing activity, which is a limitation of this research. To address this gap, future research, including VN evaluations, will be conducted to comprehensively assess the humoral immune responses elicited by our cell-based vaccine platform. Although the enhanced HI antibody titers elicited by the cell-based vaccine indicate promising humoral immunogenicity, a limitation of this study is the absence of a live-virus challenge experiment. Therefore, future preclinical challenge studies are essential to definitively confirm the protective efficacy of the developed SIV vaccine against virulent virus infection.

In conclusion, we established a scalable, robust production process for a trivalent SIV vaccine using suspension-adapted MDCK cells in a customized serum-free medium. This platform elicited superior immunogenicity than traditional egg-based controls, even at lower nominal antigen payloads. Overall, this suspension MDCK cell-based framework serves as a reliable, efficient, and commercially viable alternative for manufacturing next-generation swine influenza vaccines.
